# We Are Family: Comparative Study of *Candida* Species and *Candidozyma auris* in Laundry (EN 17658) and Surface (Biofilm) Disinfection

**DOI:** 10.3390/pathogens15030313

**Published:** 2026-03-13

**Authors:** Britta Brands, Nicole van Leuven, Dirk Bockmühl

**Affiliations:** 1 Dr. Brill + Prof. Bockmühl GmbH Institute for Applied Hygiene, 47533 Kleve, Germany; britta.brands@bb-appliedhygiene.com; 2 Faculty of Life Sciences, Rhine-Waal University of Applied Sciences, Marie-Curie-Str. 1, 47533 Kleve, Germany; nicole.vanleuven@hochschule-rhein-waal.de

**Keywords:** *Candidozyma auris*, biofilm, surface disinfection, hygiene, *Candida*

## Abstract

The rising prevalence of *Candidozyma auris* and *Candida parapsilosis*, characterized by high surface persistence and biofilm-forming capabilities, challenges the efficacy of standard laundry and surface disinfection protocols. This study evaluated the effectiveness of laundry processes according to EN 17658 at 20 °C, 30 °C and 40 °C and two surface disinfectants (bead assay for biofilms) against two *Candida albicans* strains, *C. parapsilosis*, and *C. auris*. Results indicated that *C. auris* is more resilient than other strains, surviving laundry treatment with activated oxygen bleach at 40 °C; maximum efficacy required a colour powder detergent supplemented with a bleach-releasing additive at 40 °C. While alcohol- and aldehyde-based surface disinfectants were effective per EN 13697 criteria, their efficacy against biofilms—tested on glass, stainless steel, polypropylene, and PTFE—was highly dependent on both the strain and the surface material. These findings demonstrate the reduced susceptibility of *C. auris* to standard laundry disinfection and highlight that biofilm eradication is a complex process influenced by strain-specific attributes and surface characteristics.

## 1. Introduction

Recently, a rise in infections by different *Candida* species, such as *Candidozyma auris* (basionym *Candida auris*) [1] and *Candida parapsilosis,* has been reported [2,3,4].

*Candida parapsilosis* has been found in dishwashers [5,6] and washing machines [7] in the domestic setting. It can persist on surfaces for up to 14 days [8,9] and has shown the ability to cause pneumonia, even in immunocompetent individuals [10].

A lot of studies have been investigating several aspects of this yeast, covering the antifungal resistance [11,12,13,14,15,16,17,18], the ability to form biofilms [17,19] and the fact that different clades have been detected and characterized [1,20,21,22].

After 2019, there have been several reports of rising number of fungal infections caused by *Candidozyma auris* and *Candida parapsilosis* [4,15,23,24,25,26,27,28,29,30] in different settings and regions.

*C. auris* was first isolated in Japan from an ear canal infection in 2009 [31], and then was detected in Asia [32], the United States [26,33], Africa [34,35] and Europe [29,36], causing infections in more than 35 countries in 2022 [14].

The present study was designed to investigate textile disinfection and surface disinfection, two procedures that play a pivotal role in the control of pathogens. It focused on the comparison of well-known strains (*Candida albicans*) and the strains that are more recently more often discovered in healthcare settings (*Candidozyma auris* and *Candida parapsilosis*), as it was unknown whether the different strains would behave similarly or differently when treated in the same way.

For the textile disinfection, this study investigated the reduction of different *Candida* species and *Candidozyma auris* in laundry tests according to EN 17658, a method that uses a lab-scale washing machine, thus allowing for standardized comparisons [37,38,39] and is designed for the domestic sector. The four strains included two different strains of *Candida albicans*, one strain of *Candida parapsilosis* and one strain of *Candidozyma auris*.

For the surface disinfection, normative suspension tests according to EN 1650 and surface tests according to EN 13697 are normally used. However, both of these tests use planktonic cells, while in a real-life setting, the cells must be assumed to be present in a more or less developed biofilm [40,41]. These tests do apply for the medical sector and the log reductions cited later are taken from these.

Therefore, we decided to use a bead assay for biofilms, described by Konrat et al. [42], where the four tested strains that have been investigated in the laundry experiments are grown as biofilms on the surface of beads of different materials under the influence of shear forces.

Apart from simulating a close-to-life situation, we aimed to evaluate whether this test system is suitable for testing yeasts on different surface materials that are of relevance in both the domestic and the healthcare sector.

Both are highly relevant not only in the domestic sector, but also in the healthcare setting, including hospitals as well as other care facilities.

## 2. Materials and Methods

### 2.1. Test Strains and Culturing

Four different test strains were received either from the German Collection of Microorganisms and Cell Cultures (DSMZ, Braunschweig, Germany) or from the American Type Culture Collection (ATCC, Manassas, VA, USA) (Table 1).

The used strain of *Candidozyma auris* (basionym *Candida auris* [1]) is identical to JCM15448, which represents the type strain and belongs to clade 2 [43].

The test strains were obtained as freeze-dried cultures from the corresponding source.

Upon receipt they were rehydrated as recommended by the supplier. The rehydrated cultures were split to prepare liquid cultures for the preparation of glycerol stocks as well as cultures on solid medium for testing. As a medium, malt extract broth or malt extract agar was used. Cultures were incubated at 30 °C for 48 h.

After incubation, portions from the liquid culture were mixed in equal volumes with 80% glycerol in screw-cap reaction tubes. These tubes were transferred to −80 °C and are kept at that temperature to generate fresh cultures from the original culture.

### 2.2. Laundry Tests According to EN 17658

We have performed laundry tests according to EN 17658. In brief, in these tests, cotton swatches are inoculated with microorganisms and are washed in a lab-scale washing machine simulator. The vessel contains textile ballast load, organic ballast load, steel balls to mimic the mechanical action in a washing machine and the product to be tested diluted in water of standard hardness. After the process, the remaining microbial load is compared to the initial load and the reduction is calculated.

We have chosen a total of five different detergents to be used in the test plus the control without detergent (Table 2). We included the International Electrotechnical Commission (IEC) base detergent, the IEC detergent with the standardized bleach system, with 13–14% activated oxygen bleach (AOB), a market available colour powder detergent (CPD) without bleach, the same colour powder detergent combined with a bleach-releasing additive (>30% AOB) and a heavy-duty powder detergent (HDD) with 5–15% AOB. This choice was made to cover the long-known IEC standard on the one hand and at the same time having a look at market-available detergents that might be used in real situations.

In Europe, detergents are often used in powder form. There are special formulations for coloured textiles that usually do not contain any bleach or only very low amounts of bleach.

Laundry tests were performed as described in EN 17658 [39] using the Rotawash, (SDL Atlas, Rock Hill, SC, USA), a lab-scale device that can hold up to 12 stainless steel vessels mounted to a rotating axis.

The vessels have a volume of 1.2 L and are filled with 100 g polycotton textile load (wfk20A, wfk Testgewebe, Brüggen, Germany) to which organic load and 8 steel balls that simulate the mechanical effects of a washing machine are added.

To this ballast textile load, 1 cm × 1 cm cotton swatches (wfk10A, wfk Testgewebe, Brüggen, Germany) that were previously boiled three times in double-distilled water, dyed using ‘Simplicol Textilfarbe Intensiv’ (Brauns Heitmann, Warburg, Germany), dried, and sterilized and then artificially inoculated with test strains are added alongside sterile cotton swatches.

Swatches of different colours contain different test organisms which are simultaneously evaluated in the same test run. The different colours are needed to distinguish between the different test strains.

Test strains were harvested from agar, washed in 0.9% NaCl, resuspended in BSA (0.3 g/L), and 30 µL of inoculum was applied to each swatch, then dried under sterile conditions.

Solutions of the tested products were prepared in water of standard hardness (WSH, 375 ppm CaCO_3_, preparation as in, e.g., DIN EN 1276 [44]). The solutions were prepared shortly before the start of the test. WSH was pre-tempered to the test temperature, and the test product was dosed according to the manufacturer’s instructions. The prepared test solution was added to the beaker. A liquor ratio of 1:6 was used for all tests equalling 600 mL of the test solution per beaker.

The prepared beakers were closed and transferred to the pre-tempered Rotawash where the mechanical action was started for the tested contact time.

Tests were performed with a contact time of 45 min and at temperatures of 20 °C, 30 °C and 40 °C. For each combination, three independent replicates were done. At the end of the contact time of 45 min, the beakers were removed from the Rotawash. A washing water sample was taken directly and neutralized. The washed carriers were retrieved and transferred to reaction tubes containing 1 mL of neutralizer (compositions of neutralizers can be found in the Supplementary Files). The remaining microorganisms were extracted from the swatches and the remaining count was determined by inoculation of decimal dilutions of the extract to agar plates. The reduction was calculated as the difference between the initial count of unwashed carriers and the remaining count of washed carriers.

The cross-contamination to sterile swatches was determined as described in EN 17658. Reductions and cross-contamination in the water control without product were determined to prove the validity of the procedure.

All results represent means ± the standard deviation of three independent repetitions.

### 2.3. Efficacy of Surface Disinfectants Against Candidozyma/Candida Biofilms

We used two surface disinfectants that have proven efficacy against *Candida albicans* in standardized tests based on EN 1650, EN 13697 and the additional requirements by VAH.

We tested one alcohol-based surface disinfectant and one aldehyde-based disinfectant and used the recommended concentration and contact time given on the respective packaging.

The biofilms were grown on beads as described for bacteria by Konrat et al. [42]. However, the incubation time was increased to 48 h, and the incubation temperature was set to 30 ± 1 °C to achieve optimal growth conditions for the used fungal strains. We have used four different bead materials: glass (Paul Marienfeld GmbH, Lauda-Königshofen, Germany), stainless steel (5 mm Lagerkugeln, Sturm Präzision GmbH, Oberndorf, Germany), polypropylene (PP) and polytetrafluoroethylene (PTFE, Kugelfertigung Hoch GmbH & Co. KG, Hassfurt, Germany).

Both PTFE and PP are used as material for catheters, implants and as stitch material [45,46,47,48,49], and in addition, PP is also used for surgical masks and as material for trays and other surfaces that are frequently disinfected due to their resistance against chemicals and as compared to stainless steel the absence of rust due to chemical oxidation. Stainless steel is often used in operating rooms, surgical instruments and for vessels due to the ease of cleaning and their longevity. Glass has been included, as windows, but also transparent sliding doors (in intensive care units), but also glass separations which are often used in entrances are made of glass and thus are part of the surfaces in hospitals which need to be cleaned regularly.

All beads have been thoroughly cleaned (sonication, isopropanol, distilled water, drying) to remove any production residues and sterilized before use. Beads are used for the assay only once. To grow the biofilms, a single bead was placed in the well of a 24-well plate containing 1 mL malt extract broth with a diluted pre-culture (diluted to contain approximately 10^5^ cells/mL). The prepared plate was incubated for 48 h at 30 ± 1 °C under constant agitation in an orbital shaker (150 rpm). After initial surface attachment of the cells from the culture, the biofilm forms on the bead surface [42].

At the end of the incubation period, each bead was taken out with tweezers and dip-washed in a well containing 2 mL sterile double-distilled water to remove any cells that were not part of the biofilm. The dip-washed beads were placed in sterile 2 mL reaction tubes for the test. The disinfectant was prepared according to the manufacturer’s instructions or used neat in case of the ready-to-use products.

Of the disinfectant, 200 µL was added on top of the bead in the reaction tube. The tube was incubated for the recommended contact time and at the recommended temperature. At the end of the contact time, 1800 µL of an appropriate neutralizer was added to the tube and mixed by inverting 5 times. After a neutralization time of 5 min at 20 °C, the tubes were transferred to an ultrasound bath.

After sonication for 10 min at 40 kHz using 200 W_eff_, the tube was mixed thoroughly again and 200 µL from each tube was transferred to a well of a 96-well plate. From this original extracted sample, a decimal dilution series in the neutralizer was prepared with a total volume of 200 µL per well. From each well, 5 µL was spotted on a gridded, square agar plate (688102, Greiner Bio-One, Frickenhausen, Germany) filled with malt extract agar (Merck, Germany) containing Chloramphenicol (Neolab, Heidelberg, Germany). The plate was incubated for a total of 7 days at 30 °C. After 48 h and then again after every additional 24 h, the colonies on the plate are counted. In case of a change, the highest number is used for the calculation of the cell count.

All results shown are means ± standard deviation of four independent repetitions.

### 2.4. Statistical Tests

All statistical tests were performed using GraphPad Prism Version 10.5.0.

For the tests according to EN 17658, Two-way ANOVA followed by Tukey’s multiple comparison testing was performed. For the bead biofilm assays, the same analysis was used to identify differences between the two tested surface disinfectants. For the influence of the test strain and the used bead material on the initial counts, Kruskal–Wallis test followed by Dunn’s multiple comparison was used.

## 3. Results

### 3.1. Laundry Tests According to EN 17658

The initial cell counts on the inoculated carriers were within one logarithmic step for all the test strains, with 7.2 for *C. parapsilosis* 5784 and *C. albicans* 1386, 7.6 for *C. albicans* SC 5314 and 7.9 for *C. auris* 21092.

**Figure 1:**
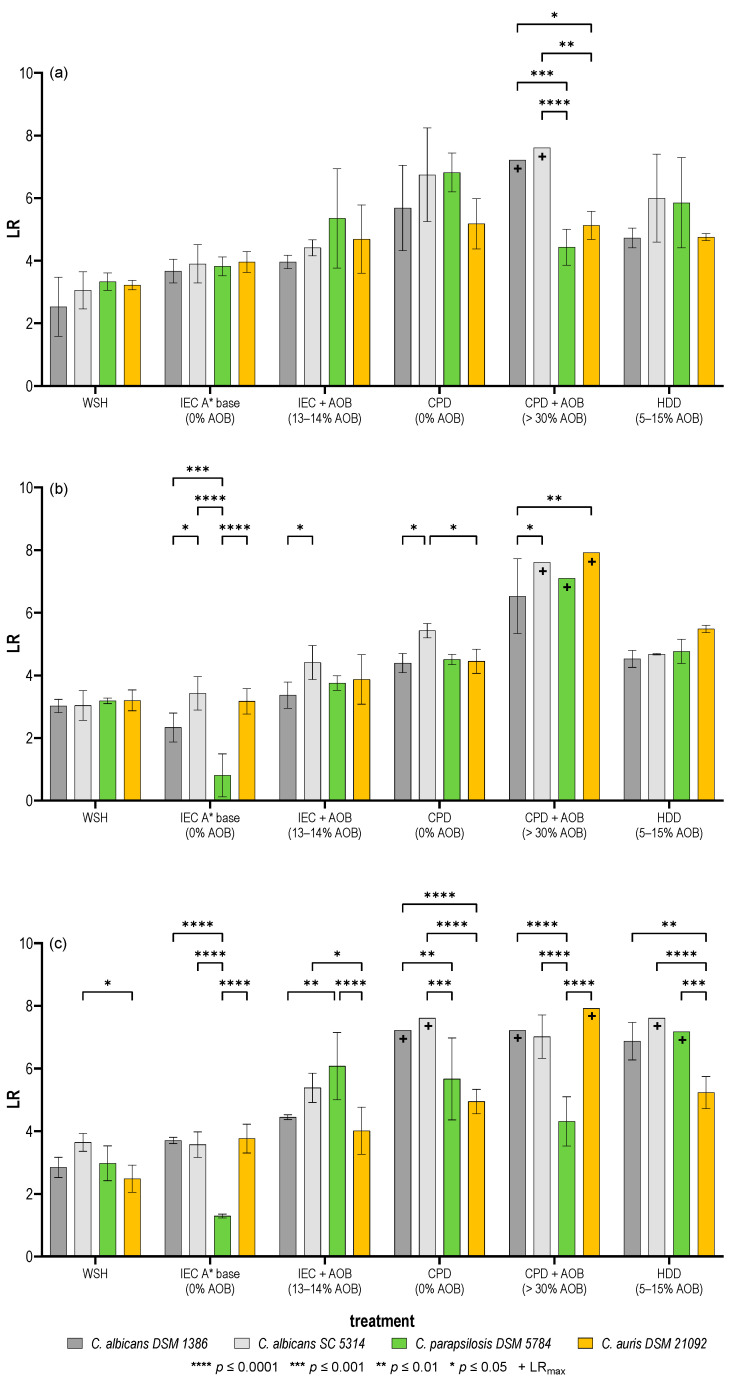
Logarithmic reductions (LR) of the different test strains after treatment of the textiles in an EN 17658 test with different detergents after a contact time of 45 min and different test temperatures. (**a**) 20 °C, (**b**) 30 °C, (**c**) 40 °C. Means ± standard deviations of three independent repetitions are shown. Asterisks indicate statistically significant differences between the test strains and the + indicates that the maximum LR was reached.

The test with water of standardized hardness (WSH) led to reductions of around 3 log steps for all tested strains and in all tested temperature settings. Only for *C. albicans* SC5314, the reduction at a temperature of 40 °C was slightly higher, reaching a reduction of 3.6 log steps which was significantly higher than the reduction in *C. auris* (Figure 1).

The addition of IEC base detergent to the test system generally increased the LR values to a range between 3.7 and 4 log with a test temperature of 20 °C and 40 °C. At a test temperature of 40 °C however, there was a remarkable decrease in the reduction in *C. parapsilosis* 5784 to 1.3 log. At the test temperature of 30 °C, the addition of the IEC base detergent did not increase the reductions but on the contrary, the reductions were reduced significantly for *C. albicans* 1386 (2.3 log) and *C. parapsilosis* 5784 (0.8 log).

The addition of a combination of the IEC base detergent and activated oxygen bleach (AOB) to the test system did increase the achieved reduction only slightly at a test temperature of 30 °C, varying from 3.4 to 4.4 log. The reduction was more uniform with these test parameters compared to the base detergent alone. Here, *C. parapsilosis* was reduced like the other test strains. At the remaining test temperatures, the reduction was higher, ranging from 4 to 5.3 log at 20 °C and 4 to 6 log at 40 °C. At both temperatures, the highest reduction was found for *C. parapsilosis* 5784, being significantly higher than for the remaining organisms at 40 °C.

The use of a market colour powder detergent (CPD) increased the reductions at all test temperatures compared to the base detergent and the base detergent combined with AOB. The achieved reductions ranged from 5.2 to 6.8 log at 20 °C, 4.4 to 5.4 log at 30 °C and 4.9 to 7.6 log at 40 °C. At 30 °C, the reductions in all test strains were slightly increased when compared to the use of IEC base detergent alone or in combination with AOB. *C. albicans* SC 5314 showed the highest reduction of 5.4 log and was significantly more reduced than the other test strains. At 20 °C, the reduction in *C. auris* 21092 was lowest of the four test strains, with the observed differences not being significant. At 40 °C, both *C. albicans* 1386 and *C. albicans* SC5314 reached the maximum logarithmic reduction. The lowest reduction was found for *C. auris* 21092.

When CPD was combined with an additional AOB-releasing additive, both *C. albicans* strains reached the maximum LR at 20 °C, while the reduction in *C. parapsilosis* 5784 and *C. auris* 21092 remained at the same level that was identified without the bleach-releasing additive. At 30 °C, the addition of the additive increased the LR to LR_max_ for three of the four test strains. Only *C. albicans* 1386 was detected in very low numbers, leading to a significantly reduced LR compared to the other test strains. At 40 °C, the additive increased the LR of *C. albicans* 1386 and *C. auris* 21092 to LR_max_. *C. albicans* SC5314 could still be detected at very low numbers, but under these conditions, the LR of *C. parapsilosis* 5784 was significantly the lowest, reaching 4.3 log.

In the test with the high-duty powder detergent (HDD), the reductions were generally lower compared to the combination of PDW and bleach-releasing additive. At 20 °C, the LRs ranged from 4.7 log for *C. albicans* 1386 and *C. auris* 21092 to 5.9 for *C. parapsilosis* 5784 and 6 for *C. albicans* SC5314. At 30 °C, the LRs were around 4.7 for both *C. albicans* strains and *C. parapsilosis* 5784, while the LR of C. auris 21092 reached 5.4 log. At the highest test temperature of 40 °C, *C. parapsilosis* 5784 and *C. albicans* SC5314 reached LR_max_. The LR of *C. albicans* 1386 was 6.9. Under these conditions, *C. auris* 21092 showed the lowest LR, reaching 5.2, which was significantly lower compared to all other test strains.

**Figure 2:**
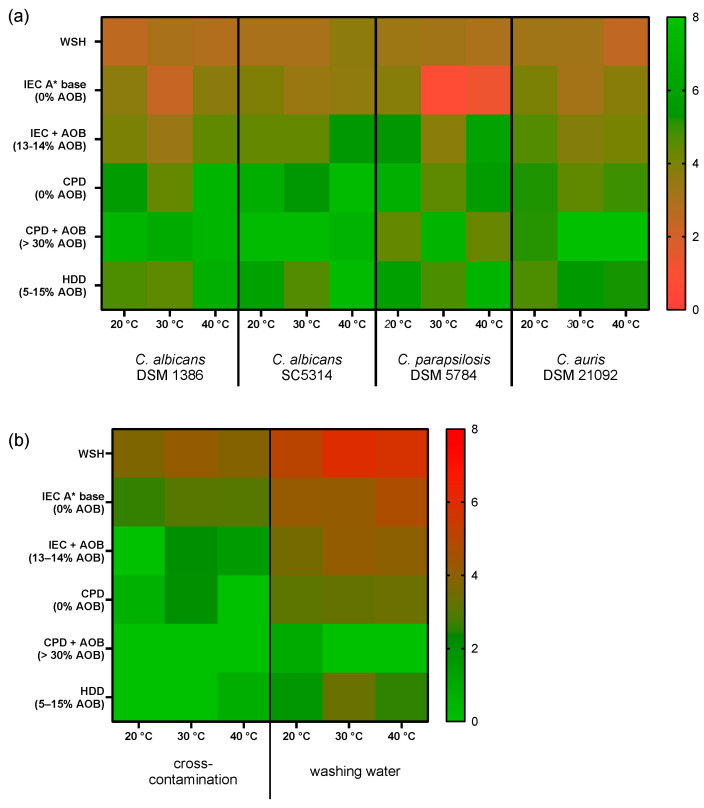
Heat map representation of the logarithmic reductions (LR) of the four test strains (**a**); cross-contamination for sterile swatches and microbial counts in the washing water (**b**) in the EN 17658 tests for different detergents and WSH at different temperatures. In part (**a**), green fields indicate higher LRs, while in part (**b**), green fields indicate lower counts. Mean values from 3 independent repetitions re shown.

Figure 2 provides a heat map summary of all the logarithmic reductions in the test organisms (Figure 2a), as well as the observed cross-contaminations and the microbial load of the washing water after the test (Figure 2b) for all tested combinations of temperatures and detergents for the four test strains.

The heat map clearly indicates that the use of detergent did reduce the microbial load on the textile and in the washing water compared to water alone except for IEC base detergent and *C. parapsilosis* 5784 as test organism. Adding AOB to the IEC base detergent further reduced the microbial load with all test organisms.

Using the tested market detergents resulted in lower microbial counts compared to the reference detergent. With the market detergents, the reductions were lower with CPD than with HDD. In most cases, the addition of the AOB-releasing additive did reduce the microbial load even further, except for *C. parapsilosis* 5784 and test temperatures of 20 °C and 40 °C.

The same effects became visible when analyzing the cross-contamination and the microbial load in the washing water. Here, the addition of detergent reduced the cross-contamination to sterile textiles at all temperatures. The test in which HDD was used showed a higher cross-contamination than the process with CPD with AOB-releasing additive at a test temperature of 40 °C.

The microbial transfer to the washing water decreased with the use of detergent and was further decreased by the use of market detergent and especially detergent with AOB. Even when HDD was used, there was still a mean microbial load of between 2 and 3 log in the washing water. With the use of CPD with the AOB-releasing additive, the microbial load in the washing water was reduced under the lower detection limit at temperatures of 30 and 40 °C. Only at 20 °C, there was a detectable remaining count in one of three independent repeats.

### 3.2. Efficacy of Surface Disinfectants Against Candida/Candidozyma Biofilms

In addition to the reduction in the various strains in washing processes with different detergents, the efficacy of various surface disinfectants against biofilms of the four test strains was investigated.

**Figure 3:**
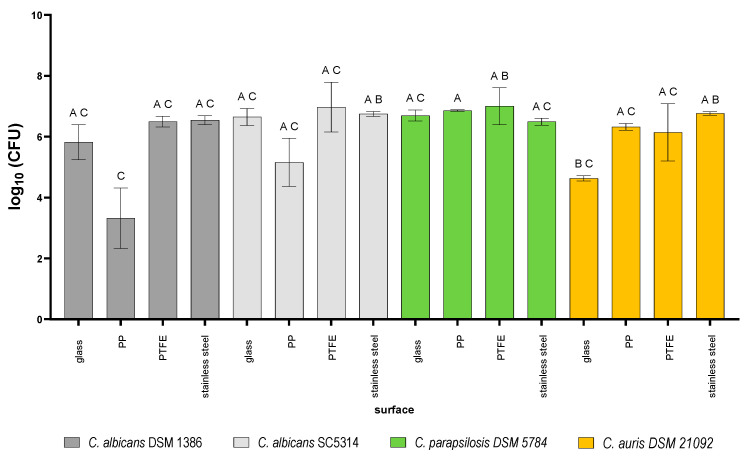
Initial counts of the four Candida/Candidozyma strains when grown as biofilms on beads of different materials. Means ± standard deviations of four independent surfaces are shown. Different letters indicate significantly different initial counts.

The four test strains were grown on different surface materials for 48 h to form a young biofilm. The initial counts of the four test strains on the different materials are shown in Figure 3.

Although most of the initial counts were in the same range between 6.5 and 7, there were some combinations of test strain and material that showed significantly different initial counts under identical growth conditions. The significance levels can be found in Supplementary Table S2 in detail.

For the well-known test strain *C. albicans* 1386, the biofilm formation was lowest on PP beads while being comparable on the other three test materials.

For *C. albicans* SC5314, the biofilm formation on PP was also reduced, but not to the extent observed with *C. albicans* 1386. *C. parapsilosis* 5784 showed the most consistent amount of biofilm on all tested materials. Here PP showed the lowest standard deviation of all test materials.

*C. auris* 21092 showed varying degrees of biofilm formation depending on the test material. The biofilm formation was lowest on glass (4.6 log), while for the remaining materials, the biofilm formation was above 6 log.

**Figure 4:**
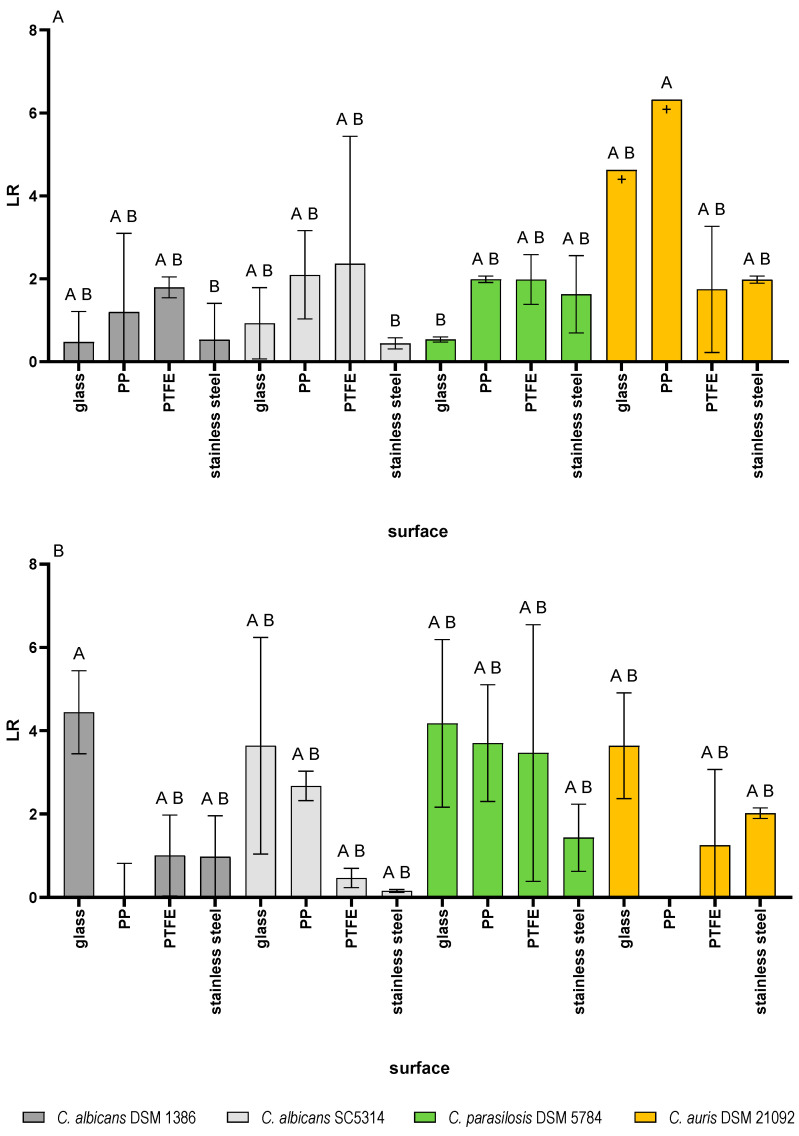
Logarithmic reductions (LR) achieved by an alcohol based (**A**) and an aldehyde-based (**B**) surface disinfectant used against biofilms grown on beads of different materials. Different letters indicate statistically significant LR-values. Means ± standard deviations of four independent surfaces are shown. + indicates that the number of recovered cells is not zero, but under the lower detection limit.

Figure 4 shows the logarithmic reduction (LR) of the four test strain biofilms on the surface after application of either the alcohol-based disinfectant (A) or the aldehyde-based disinfectant (B).

When the biofilms were treated with an alcohol-based disinfectant, the four test strains showed a differentiated behaviour. The highest LR of 6.3 was achieved on PP for *C. auris* 21092, followed by the LR of *C. auris* on glass (4.6), while the LRs of *C. auris* on PTFE and stainless steel both were around 2.

*C. parapsilosis* 5784 was reduced by about 2 log steps on PP, PTFE and stainless steel, while the reduction on glass was remarkably lower (0.5 log).

For *C. albicans* 1386 the reduction on glass and stainless steel were lowest (0.5 log). The reductions on PP and PTFE reached 1.2 and 1.8 respectively. The reduction pattern of the other *C. albicans* strain, SC5314, was similar in most cases with the lowest LR being detected on stainless steel (0.4), followed by glass (0.9), PP (2.1) and the highest reduction of 2.4 on PTFE.

The treatment of the biofilms with an aldehyde-based disinfectant showed different reduction patterns compared to the alcohol-based product. Here, for all test strains, the highest LR was observed on glass (4.4 for *C. albicans* 1386, 4.2 for *C. parapsilosis* 5784, 3.6 for *C. albicans* SC5314 and *C. auris* 21092).

On PP, no reduction was observed for *C. albicans* 1386 and *C. auris* 21092, while *C. albicans* SC 5314 and *C. parapsilosis* 5784 showed clear LRs of 2.7 and 3.7 respectively. The reduction on PTFE was lowest for *C. albicans* SC5314 (0.5). For *C. albicans* 1386 (1) and *C. auris* 21092 (1.25), the reductions were slightly higher, while the LR was most pronounced on PTFE for *C. parapsilosis* 5784, reaching 3.5.

On stainless steel, *C. auris* 21092 showed the highest reduction (2), followed by *C. parapsilosis* 5784 (1.4) and *C. albicans* 1386 (1). *C. albicans* SC5314 showed the lowest LR with 0.15.

**Figure 5:**
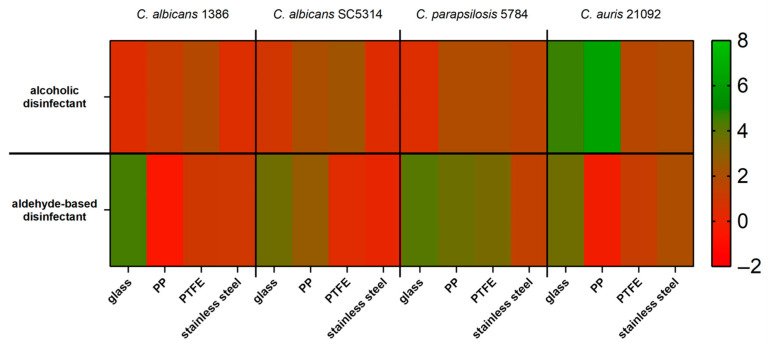
Heat map representation of the logarithmic reductions (LR) of the four test strains on different surfaces and after treatment with either an alcoholic (**top**) or aldehyde-based (**bottom**) surface disinfectant. Low LRs are indicated in red, high LRs in green (see scale on the right). Mean values from four independent surfaces are shown.

Figure 5 summarizes the reductions in the four test strains on the different surface materials after treatment with either the alcoholic disinfectant (top) or the aldehyde-based disinfectant (bottom). The reductions were analyzed in a Tukey multiple comparisons test; the corresponding results can be found in Supplementary Table S3.

For each of the test strains, there were some differences in the reductions based on the used disinfectant. The difference for *C. albicans* 1386 was most pronounced on glass surfaces. While the LR with the alcoholic disinfectant was around 0.5, the LR with the aldehyde-based disinfectant was 4.5. On stainless steel, the reduction with the aldehyde-based disinfectant was also higher than with the alcohol-based product. For the remaining two test surfaces, the reductions with the alcoholic surface disinfectant were higher than with the aldehyde-based product.

Similar differences could also be observed for *C. albicans* SC5314. While both disinfectants reduced the biofilms equally on PP and stainless steel (although the total reduction on PP was higher than on stainless steel), there were clear differences on glass and PTFE surfaces. On the glass surface, the alcoholic disinfectant led to a lower LR than the aldehyde-based disinfectant (approx. 1 and 3.6, respectively). This was similar on PTFE, where the alcoholic and the aldehyde-based disinfectant reached an LR of 2 and 2.7, respectively.

*C. parapsilosis* 5784 was reduced similarly by both disinfectants when grown on stainless steel. On the three remaining surfaces, the LRs achieved by the aldehyde-based disinfectant were higher than with the alcoholic disinfectant. The difference between the two was most pronounced on the glass surface on which the alcoholic disinfectant caused an LR of 0.5, while the aldehyde-based disinfectant reached an LR of 4.2.

*C. auris* 21092 showed similar reductions by both disinfectants on three of the surfaces. On PP, however, the difference was most pronounced. While the aldehyde-based disinfectant did not reduce the biofilm, the alcoholic disinfectant achieved an LR of 6.

The overall presentation in the heat map in Figure 5 reveals that the reductions differed within the two groups of used disinfectant depending on the surface type and the test strain as well as for the same test strain and surface based on the used disinfectant.

## 4. Discussion

The obtained results clearly show that there are major differences in the antimicrobial susceptibility of different Candida strains in the investigated settings, which will be separately discussed due to their different nature.

### 4.1. Laundry Tests

It has been described in the literature that *Candida albicans* can survive and proliferate on textiles and is transferred during laundering [50]. The fact that *C. parapsilosis* could be isolated from different surfaces, including washing machines, suggests that this strain might also be transferred in the washing process if not properly reduced [51]. As, to our knowledge, data on the reductions in the four test strains are not publicly available, the discussion focuses on possible explanations of our findings based on related literature.

The initial counts of the *Candida* strains on cotton swatches ranged between 7.17 and 7.92 and thus do not differentiate significantly from each other. Likewise, the reductions in WSH were not significantly different both at 20 °C and 30 °C, suggesting that the removal of the different test strains in water combined with the applied mechanical action might be similar, although there is a slightly higher remaining count of *C. auris* 21092 in both settings. At a test temperature of 40 °C however, the reduction in *C. auris* 21092 was significantly lower than for the other test strains, resulting in a higher remaining count on the treated textiles. This is in line with the fact that *C. auris* may be well adapted to higher temperatures, which is one of the hypotheses why this pathogen is emerging coincidently with global warming [52]. Similarly, the addition of detergents revealed that *C. auris* 21092 was the test strain with the highest remaining numbers at the end of the laundry process. Whether this is due to the up-regulated cell surface adhesins [53,54,55,56] or other factors remains unclear at this point, especially as the processes are not yet fully understood.

It is well known that the wash cycle time, the temperature and the detergent formulation do have an influence on the reduction in *C. albicans* in the laundering process [57]. This has been confirmed in this present study and is also within limits true for the other tested *Candida* strains.

*C. auris* 21092 proved to be the most resistant of the test strains when regular powder heavy-duty detergent was used at a wash temperature of 40 °C. Here, *C. auris* 21092 was reduced by about 5 log, while both *C. albicans* strains were reduced by about 7 log steps. This reduction in *C. albicans* was comparable to the results found in a domestic washing machine described by Honisch et al. [58].

These findings suggest that (i) the obtained reductions in EN 17658 are transferable to the results in domestic washing machines in general and that (ii) *C. auris* 21092 is more resistant even when an AOB-containing heavy-duty powder detergent is used, which is recommended in case of fungal infections together with elevated temperatures [59,60,61].

When colour powder detergent was used in combination with an additional bleach-releasing additive, in which the amount of activated oxygen bleach is more than twice as high as in the normal heavy-duty powder detergent, the reduction in the four test strains and especially *C. auris* 21092 could be enhanced at a washing temperature of 40 °C and also the cross-contamination was greatly reduced using this combination.

Temperature alone did not cause significantly higher LRs when comparing the LRs of a single test strain (see Supplementary Figure S1).

The detected differences between the strains might not always be significant as the standard deviations for some strains are higher than for others. During the development, the method has shown a high reproducibility of results, which was supported by an international ring trial [38]. Experiences from the daily lab routine have shown that this is true, but that on the edge of efficacy, the standard deviations often show bigger ranges (unpublished data). Likewise, some phenomena remain poorly understood, e.g., a higher reduction at lower temperatures. This might apply for at least some of the strains included in this trial. Future additional research will help to better understand the differences between the test strains and help to clear the picture that has now been gained in this first comparative test setup.

Future research might also show whether the current criteria for a yeasticidal claim, including the reduction on the contaminated carriers of at least 3 log steps, combined with the limit for cross-contamination to sterile carriers (≤1.54 log) and the limitation of the microbial load in the water (≤1.15 log) need to be revised.

### 4.2. Biofilm Assay

Two disinfectants that are approved for use in hospital and healthcare settings in Germany have been tested and have proven to be effective against the commonly used test strain *C. albicans* 1386 in two independent laboratories according to VAH regulations [62].

**Figure 6:**
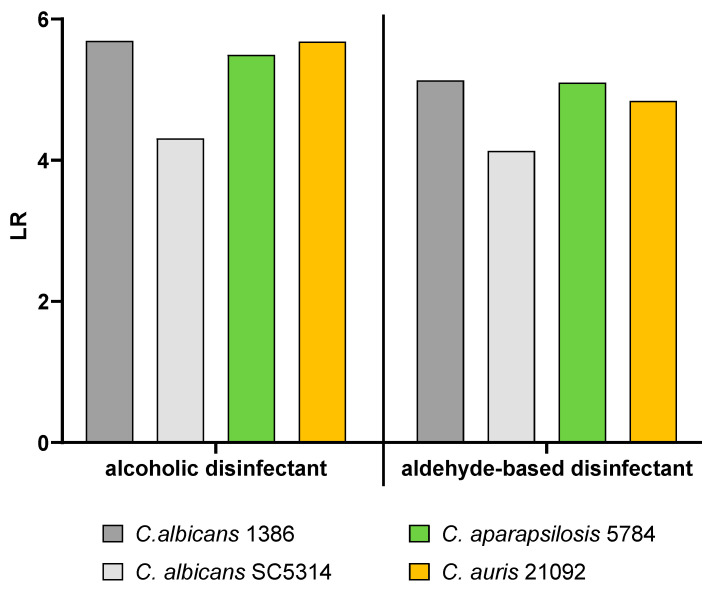
Logarithmic reduction (LR) of different Candida test strains achieved by use of an alcoholic (**left**) or aldehyde-based disinfectant (**right**) in a test according to EN 13697. Bars are weighted averages of two determinations and thus show no error bars.

To show the general antimicrobial susceptibility of the tested *Candida* strains, we performed surface disinfection tests based on EN 13697 [63]. As there are currently no criteria to evaluate an efficacy against biofilms on surfaces, the standard surface disinfection criteria are the best reference available. In these tests, the logarithmic reductions ranged between 4 and 6 log (Figure 6) thus meeting the normative requirements for antifungal surface disinfectants that are used without mechanical action (logarithmic reduction > 3).

This is in line with recent studies, which investigated the efficacy of surface disinfectants with different active agents against *C. auris* and other *Candida* species that were inoculated to surfaces from cultures [17,23,64] and also against dry surface biofilms [65], suggesting a tendentially lower susceptibility of *C. auris* and its ability to form persistent biofilms [66].

Although the two disinfectants exhibited a sufficient efficacy against the tested *Candida* strains in the carrier tests, the two disinfectants showed different levels of reduction against the test strains grown as biofilms as can be seen in Figure 5.

There are differences between the test strains on the same surface, differences between different surfaces for the same test strain and also differences between the two disinfectants for the same strain or the same surface. The matrix of significances between all tested combinations is given as Supplementary Table S3.

This matrix reveals that for *C. albicans* SC5314 there are no significant differences when comparing the different test surfaces or the two different disinfectants.

For *C. parapsilosis* 5784, the only significant difference observed with the use of this test strain was found on glass and was due to the use of the two different disinfectants.

For the commonly used test strain *C. albicans* 1386, there were a few significant differences. One was observed on the glass surface using different disinfectants. The second one was observed between the glass surface and the PP surface when the aldehyde-based disinfectant was used, thus due to the test surface. The third difference was observed between two different surfaces and different disinfectants and seems to be due to the combination of test surface and disinfectant.

For *C. auris* 21092, more significant differences between conditions could be observed. All of them had in common that the comparison included a PP surface. In the combination with the alcoholic disinfectant, there were significant differences in the LR to the PP surface with the aldehyde-based disinfectant and, as well as the PTFE, the stainless-steel surfaces, no matter with which disinfectants those were treated.

When the PP surfaces were treated with the aldehyde-based disinfectant, there were significant differences to the glass surface, independent of the disinfectant that was used on glass.

The significance matrix not only shows significances for comparisons within the same test strain, but also the combination of different test strains combined with the surfaces and the disinfectant. This matrix shows that there are differences not between all combinations, so there are certain combinations of strain, surface and disinfectant that lead to these differences.

*C. auris* 21092 shows many significant differences to the other test strains, especially when *C. auris* 21092 was tested after growth on PP surfaces. It has been shown that *C. auris* does contain the SCF1 gene that plays a crucial role in cell attachment to polymer surfaces [55], so this could be at least part of the explanation for the observed differences. Why this was only observed on PP and not PTFE as well remains unclear.

Malavia-Jones et al. have described that the aggregation of *C. auris* is strain and temperature dependent with higher aggregation and formation of extracellular matrix proteins at higher growth temperatures [67]. Given this, the biofilms used in this study might be an over-estimation of the actual biofilm formation at room temperature. However, it has been reported that *C. auris* and other *Candida* species persist on dry or moist surfaces for up to seven days [64] and thus have generally more time to form biofilms than in our experimental setup. It has been shown that mature *C. auris* biofilms show increased transcription of cell wall genes and especially in intermediate and mature stages, several efflux pump genes are significantly increased [68]. It remains unclear if longer incubation times of the forming biofilms would have further increased the resistance of the four test strains to the tested surface disinfectants. This would be an interesting point for future research.

Based on the findings by Malavia-Jones et al. [67] and Kean et al. [68], it is likely that the efficacy of yeasticidal disinfectants differs with biofilms formed under different temperature conditions and with different biofilm ages. With these results, one could develop a cleaning regime that would cover the best practice for the prevention of *Candida* biofilms depending on the actual growth conditions.

## 5. Conclusions

We have shown that four different test strains of the genera *Candida* and *Candidozyma* do show differential behaviour in laundry tests according to EN 17658 as well as in disinfection tests based on the bead assay for biofilms described by Konrat et al. [42]. In a laundering process with commercially available powder detergents, *C. auris* 21092 has been shown to be the most resistant strain at a washing temperature of 20 °C even with the addition of activated oxygen bleach.

An elevated washing temperature of 30 °C only has a beneficial effect if a bleach-releasing additive was added. Although the increase to a washing temperature to 40 °C led to an increase in reductions in both *C. albicans* test strains (1386 and SC 5314) as well as *C. parapsilosis* 5784, the fourth test strain, *C. auris* 21092, was not further reduced compared to 30 °C when the heavy-duty powder detergent was used. Interestingly, the market detergents perform better than the reference detergent especially at low temperatures.

The bead–biofilm assay revealed that the tested disinfectants are not able to effectively reduce all four test strains when grown as biofilms, although they pass the criteria of the standard EN 13697 test on surfaces. The four test strains are not affected by the disinfectants in the same way and the actual reduction is based on the strain itself as well as the surface it has grown on.

Thus, to treat *Candida* biofilms, it is of utmost importance to take the possible strain into consideration but also makes it necessary to treat different surface materials differently.

## Figures and Tables

**Table 1:** Overview of the test strains and their corresponding collection numbers. The collection numbers with the superscript T identify the type strains.

Test Strain	Collection Number	Source
*Candida albicans*	DSM 1386	DSMZ
*Candida albicans* SC 5314	MYA-2876	ATCC
*Candida parapsilosis*	DSM 5784 ^T^	DSMZ
*Candidozyma auris* (basionym: *Candida auris*)	DSM 21092 ^T^	DSMZ

**Table 2:** Overview of used detergents for the tests according to EN 17658. The first column shows the label used throughout this paper; the second column shows the amounts of all used components. The given dosage is based on the liquor ratio of 1:6. The INCI list for all detergents is provided in the Supplementary Files.

Label	Composition/Reference	Dosage
WSH	Water of standard hardness (375 ppm CaCO_3_)—no detergent	600 mL
IEC A* base	Base detergent type A* IEC 60456 ed. 5 AMD1	2.460 g
IEC + AOB	Base detergent type A* IEC 60456:2024	2.460 g
	Tetraacetylethylenediamine IEC 60456:2024	0.096 g
	sodium percarbonate IEC 60456:2024	0.640 g
CPD	Market colour detergent (for INCI list see Supplementary File)	5.600 g
CPD + AOB	Market colour detergent	5.600 g
	Market Bleach releasing additive (for INCI list see Supplementary File)	3.156 g
HDD	Market heavy-duty detergent (for INCI list see Supplementary File)	5.600 g

## Data Availability

The data presented in this study are available on request from the corresponding author.

## Supplementary Materials

The following supporting information can be downloaded at: https://www.mdpi.com/article/10.3390/pathogens15030313/s1. Figure S1: Comparison of the reduction in the laundry experiments for different test temperatures and with different detergents (WSH without detergent). Means and standard deviations of three independent repetitions are shown. Different capital letters indicate that the corresponding bars are significantly different from each other. Excel file containing Tables S1–S3 on different sheets. Table S1: INCI lists of the used market detergents as provided by the manufacturer and composition of the IEC A* reference detergent. Table S2: Significance levels calculated in the statistical analysis on the initial counts of the four test strains on the different surfaces. The abbreviation ns indicates that there is no significant difference between the two compared conditions. Table S3: Significance levels calculated in the statistical analysis on the LR values of the four test strains on the different surfaces. The abbreviation ns indicates that there is no significant difference between the two compared conditions.

## Abbreviations

The following abbreviations are used in this manuscript:

AOB	activated oxygen bleach
ATCC	American Type Culture Collection
CPD	colour powder detergent
DSMZ	Deutsche Sammlung von Mikroorganismen und Zellkulturen (German Collection of Microorganisms and Cell Cultures)
HDD	heavy duty powder detergent
IEC	International Electrotechnical Commission
LR	logarithmic reduction (factor)
PP	polypropylene
ppm	parts per million
PTFE	polytetrafluoroethylene
WSH	water of standardized hardness
